# Immune status affects the clinical features and outcomes of adult patients with respiratory adenovirus infection

**DOI:** 10.1017/S0950268821002272

**Published:** 2021-10-21

**Authors:** Handan Zhao, Minghan Zhou, Qing Zheng, Guanjing Lang, Lijun Xu

**Affiliations:** 1 National Clinical Research Center for Infectious Diseases, the First Affiliated Hospital, College of Medicine, Zhejiang University, Qingchun Rd., Hangzhou, China; 2 The State Key Laboratory for Diagnosis and Treatment of Infectious Diseases, the First Affiliated Hospital, College of Medicine, Zhejiang University, Qingchun Rd., Hangzhou, China; 3 College of Medicine, Zhejiang University, Yuhangtang Rd., Hangzhou, China

**Keywords:** Clinical feature, immunocompetent, immunocompromised, outcome, respiratory adenovirus infection

## Abstract

The differences in the clinical features and outcomes of respiratory adenovirus infection (RAI) between immunocompetent and immunocompromised adult patients remain unclear. Thirty-nine adult RAI patients, including 28 (71.8%) immunocompetent patients and 11 (28.2%) immunocompromised patients were enrolled in this retrospective study. Demographic characteristics, symptoms, laboratory tests, radiographic findings, therapies and clinical outcomes were compared between the two groups. We found fever (94.9%), cough (66.7%) and sputum production (56.4%) were the most frequent symptoms. Compared with immunocompetent RAI patients, the immunocompromised RAI patients were more likely to experience anaemia (g/l; 90.8 ± 24.0 *vs* 134.3 ± 14.6, *P* < 0.001), thrombocytopaenia ( × 10^9^/l; 116.9 ± 92.7 *vs* 178.4 ± 74.6, *P* = 0.037), hypoalbuminaemia (g/l; 29.6 ± 5.5 *vs* 36.9 ± 5.2, *P* < 0.001), hyponatraemia (mmol/l; 134.8 ± 5.6 *vs* 138.5 ± 3.9, *P* = 0.026) and low levels of cholinesterase (U/l; 2650.5 ± 1467.4 *vs* 5892.8 ± 1875.1, *P* < 0.001). Chest computed tomography (CT) scans indicated that lung infiltrate was the most common finding (64.1%). Immunocompromised patients had a higher likelihood of bilateral lung involvement (72.7%) and lower lobe involvement (81.8%) of both lungs. The hospitalized mortality rate was 27.3% in immunocompromised RAI patients, but no death occurred among immunocompetent RAI patients (*P* = 0.018). Our data suggested immunocompromised RAI patients had worse laboratory test results, more bilateral lung and lower lobe involvement and higher in-hospital mortality compared with immunocompetent RAI patients.

## Introduction

Adenovirus is a double-stranded DNA virus that most often involves the respiratory tract, pharynx, conjunctiva and gastrointestinal tract [1]. Owing to a lack of humoral immunity, children <4 years old present with more than 80% of adenovirus infection cases in clinical practice [2]. Alternatively, severe adenovirus pneumonia and disseminated adenovirus infection can occur among immunocompromised adult patients [2, 3]. Occasionally, it is transmissible among healthy people living in close and crowded settings, such as college students and military recruits [2].

To date, the differences in the clinical features of RAI between immunocompromised and immunocompetent patients are still controversial. A recent study based on a small population indicated that there was no difference in clinical manifestations or the rate of severe infection between immunocompetent and immunocompromised patients [4]. However, some data demonstrated that RAI was a frequent cause of high morbidity and mortality among immunocompromised patients, with reported fatality rates reaching up to 55% [1, 5, 6]. Considering the rare and divergent data of RAI, we conducted this retrospective study to outline the clinical features and compare the outcomes of RAI between patients with immunocompetent and immunocompromised status.

## Methods

### Patient enrolment

Between January 2010 and December 2020, 136 RAI patients were admitted to the First Affiliated Hospital, College of Medicine of Zhejiang University, Hangzhou, China. Among those, 94 patients aged less than 18 years old and three patients without diagnostic information were excluded. Finally, 39 adult patients aged 18–89 years old were included in the present study. Their information (age, sex, clinical manifestations, days of hospitalisation, underlying diseases, significant symptoms, blood test results, imaging examination results, treatments and clinical outcomes) was recorded in the electronic medical record system (EMRS). Patients were followed from the first day of admission to the day of discharge.

### Diagnosis criteria

The diagnosis of RAI was confirmed based on the following criteria [2, 3, 6–8]: (1) positive symptoms of adenovirus infection; (2) a positive polymerase chain reaction (PCR) for adenovirus in respiratory samples [nasopharynx, throat swabs or bronchoalveolar lavage fluid (BALF)] or a more than four-fold increase in serum antibody titre in the convalescence period compared to that in the acute period; (3) chest radiological examination showing lesions of viral pneumonia in the lung; (4) no improvement in clinical symptoms or laboratory results after antibacterial treatment.

The severity of RAI was evaluated with the CURB-65 score, which was recommended by the British Thoracic Society. The evaluation form assigns points based on five clinical indicators ① confusion, ② urea>7 mmol/l, ③ respiratory rate≥ 30/min, ④ blood pressure (systolic (<90 mmHg) or diastolic (<60 mmHg)) and ⑤ age≥ 65 years. One point is given when in line with each indicator (range 0–5 points). A total point score≥ 3.0 was defined as severe pneumonia [9].

Immunocompromised patients represented with those: neutropenia, active solid organ or haematological malignancy, solid organ or haematopoietic stem cell transplant, uncontrolled diabetes, liver cirrhosis, significant immune deficiency (such as hypogammaglobulinaemia), human immunodeficiency virus (HIV) infection with CD4 counts<200 cells/μl or on-going treatment with chemotherapy, immune suppressive therapy (such as methotrexate, tacrolimus) or long-term (>3 months) or high-dose corticosteroids (>0.5 mg/kg/day) for more than 5 days [4, 10].

### Statistical analyses

Means ± standard deviation was used to describe continuous normally distributed variables, while medians (interquartile ranges, IQRs) were applied to describe continuous nonnormally distributed variables. Differences in categorical variables between the two groups were compared by *χ*^2^ analysis or Fisher's exact test, whereas quantitative variables were compared using Student's *t*-test or a nonparametric test, depending on whether they had a normal distribution. *P* < 0.05 (two-tailed) was defined as statistically significant. All of the data were analysed with SPSS version 22.0 (IBM, Armonk, NY, USA).

### Ethical approval of the study protocol

This study protocol was conducted in accordance with the 1975 Declaration of Helsinki and was approved by the Ethics Committee of the First Affiliated Hospital, College of Medicine, Zhejiang University (Hangzhou, China) (No. 2021-249). All data analysed were anonymous.

## Results

### Demographic characteristics and clinical features of 39 patients with clinical symptoms

In all, 100% (39/39) patients had a positive PCR for adenovirus in respiratory samples and 2.6% (1/39) had positive Ig-M in serum additionally. Of 39 patients, 69.2% (27/39) were male. Their mean age was 41.4 ± 16.8 years old. There were 71.8% (28/39) immunocompetent and 28.2% (11/39) immunocompromised patients. Namely, 5.1% (2/39) of patients had liver cirrhosis, 12.8% (5/39) had HIV infection, 5.1% (2/39) had diabetes mellitus, 2.6% (1/39) had chronic kidney disease, 2.6% (1/39) had solid organ transplantation, 5.1% (2/39) had autoimmune disease, 5.1% (2/39) had a chronic haematological disease, 2.6% (1/39) had structural lung disease, 2.6% (1/39) had cardiovascular disease, 12.8% (5/39) had blood and solid organ tumours, 12.8% (5/39) had long-term steroid use and 15.4% (6/39) used long-term immunosuppressive agents.

No significant difference was observed in sex or age between the two groups (*P* = 1.000, *P* = 0.295, respectively). The hospitalization stay was 14.0 (10.0–22.0) days in the immunocompromised group and 7.0 (5.0–9.0) days in the immunocompetent group (*P* = 0.049). Additionally, there were 36.4% (4/11) immunocompromised patients and 28.6% (8/28) immunocompetent patients with smoking habits of more than 3 years (*P* = 0.709). (Table 1).

**Table 1.** Demographic characteristics and clinical features of 39 patients with adenovirus infection in different immune status

Factors		All patients	Immunocompromised *N* = 11 (28.2%)	Immunocompetent *N* = 28 (71.8%)	*P* value
Sex (Male %)		27 (69.2)	8 (72.7)	19 (67.9)	1.000
Age (years)		41.4 ± 16.8	45.9 ± 18.5	39.6 ± 16.1	0.295
Stay of hospital (days)		8.0 (6.0–14.0)	14.0 (10.0–22.0)	7.0 (5.0–9.0)	0.049
Smoking habit (*n* (%))		12 (30.8)	4 (36.4)	8 (28.6)	0.709
Predisposing condition (*n* (%))					
Liver cirrhosis		2 (5.1)	2 (18.2)	0 (0)	–
Acquired immune deficiency syndrome		5 (12.8)	5 (45.5)	0 (0)	–
Diabetes mellitus		2 (5.1)	2 (18.2)	0 (0)	–
Chronic kidney disease		1 (2.6)	1 (9.1)	0 (0)	–
Solid organ transplantation		1 (2.6)	1 (9.1)	0 (0)	–
Autoimmune diseases		2 (5.1)	2 (18.2)	0 (0)	–
Chronic haematological disease		2 (5.1)	2 (18.2)	0 (0)	–
Structural lung disease		1 (2.6)	1 (9.1)	0 (0)	–
Cardiovascular disease		1 (2.6)	1 (9.1)	0 (0)	–
Malignancy		5 (12.8)	4 (36.4)	0 (0)	–
Steroid usage		5 (12.8)	5 (45.5)	0 (0)	–
Immunosuppressive reagent		6 (15.4)	6 (54.5)	0 (0)	–
Clinical symptoms (*n* (%))					
Fever		37 (94.9)	9 (81.8)	28 (100.0)	0.074
Cough		26 (66.7)	6 (54.5)	20 (71.4)	0.453
Sputum production		22 (56.4)	5 (45.5)	17 (60.7)	0.482
Dyspnoea		10 (25.6)	4 (36.4)	6 (21.4)	0.424
Diarrhoea		11 (28.2)	5 (45.5)	7 (25.0)	0.368
Vomiting		4 (10.3)	1 (9.1)	3 (10.7)	0.687
Sore throat		12 (30.8)	1 (9.1)	11 (39.3)	0.068
Dizzy		2 (5.1)	0	2 (7.1)	0.510
Headache		4 (10.3)	0	4 (14.3)	0.249
Myalgia		8 (20.5)	0	8 (28.6)	0.078
Sclera congestion		2 (5.1)	0	2 (7.1)	1.000

Fever (94.9%), cough (66.7%) and sputum production (56.4%) were the most frequent symptoms in our study. The incidence of fever was 81.8% (9/11) in immunocompromised patients and 100% (28/28) in immunocompetent patients (*P* = 0.074). Other atypical manifestations included sore throat (30.8%), diarrhoea (28.2%), dyspnoea (25.6%), myalgia (20.5%), etc. All of these symptoms had no statistical significance between the two RAI groups (Table 1).

### Laboratory results

We found that the haemoglobin concentration of 134.3 ± 14.6 g/l in immunocompetent patients was significantly higher than 90.8 ± 24.0 g/l in immunocompromised patients (*P* < 0.001). The platelet count in immunocompetent patients ((178.4 ± 74.6) × 10^9^/l) was also slightly higher than that in immunocompromised patients ((116.9 ± 92.7) × 10^9^/l, *P* = 0.037). Moreover, compared to total protein levels of 65.4 (58.2–69.9) g/l, albumin levels of 36.9 ± 5.2 g/l, cholinesterase levels of 5892.8 ± 1875.1 U/l and serum sodium levels of 138.5 ± 3.9 mmol/l in the immunocompetent group, the total protein levels were 58.4 (50.3–63.0) g/l, the albumin levels were 29.6 ± 5.5 g/l, the cholinesterase levels were 2650.5 ± 1467.4 U/l and the serum sodium levels were 134.8 ± 5.6 mmol/l in the immunocompromised group (*P* = 0.013, *P* < 0.001, *P* < 0.001 and *P* = 0.026, respectively). No significant differences were found in the white blood cell count, the levels of C-reactive protein, the levels of alanine aminotransferase, etc. Concomitant microorganisms including bacteria, viruses and fungi in respiratory samples were observed. There were 54.5% (6/11) immunocompromised patients and 14.3% (4/28) immunocompetent patients had other microbiological findings other than adenovirus (*P* = 0.017), although bacteria, virus or fungi had no statistical significance on distribution in each subgroup (*P* = 0.562, *P* = 0.187, *P* = 0.1125, respectively) (Table 2).

**Table 2.** Laboratory results, chest radiographic characteristics, treatment and clinical prognosis of 39 patients infected with adenovirus

	All patients	Immunocompromised *N* = 11 (28.2%)	Immunocompetent *N* = 28 (71.8%)	*P* value
Laboratory results				
WBC ( × 10^9^/l)	5.3 (4.0–9.6)	4.9 (3.2–6.0)	6.3 (4.2–9.7)	0.104
Hb (g/l)	122.1 ± 26.4	90.8 ± 24.0	134.3 ± 14.6	<0.001
Plt ( × 10^9^/l)	161.1 ± 83.7	116.9 ± 92.7	178.4 ± 74.6	0.037
CRP (mg/l)	50.5 (25.8–121.2)	58.8 (28.3–124.8)	48.7 (23.8–127.0)	0.925
PCT (ng/ml)	0.1 (0–0.3)	0.1 (0–1.0)	0.1 (0–0.3)	0.899
ALT (U/l)	40.0 (20.0–71.5)	28.0 (20.0–51.0)	40.5 (16.3–72.3)	0.851
AST (U/l)	43.0 (21.5–65.0)	40.0 (22.0–71.0)	43.5 (18.8–58.5)	0.417
TP (g/l)	63.0 (56.7–68.7)	58.4 (50.3–63.0)	65.4 (58.2–69.9)	0.013
Albumin (g/l)	34.9 ± 6.2	29.6 ± 5.5	36.9 ± 5.2	<0.001
Che (U/l)	4978.3 ± 2280.8	2650.5 ± 1467.4	5892.8 ± 1875.1	<0.001
Cr (μmol/l)	68.0 (58.0–79.5)	78 (60.0–136.0)	65.5 (54.3–77.8)	0.072
Na (mmol/l)	137.4 ± 4.7	134.8 ± 5.6	138.5 ± 3.9	0.026
Other respiratory infection (*n* (%))				
Bacteria	4 (10.3)	2 (18.2)	2 (7.1)	0.562
Virus	3 (7.7)	2 (18.2)	1 (3.6)	0.187
Fungi	5 (12.8)	3 (27.3)	2 (7.1)	0.125
Total	10 (25.6)	6 (54.5)	4 (14.3)	0.017
Chest radiographic characteristics				
Lung infiltrate (*n* (%))	25 (64.1)	10 (90.9)	15 (53.6)	0.060
Right lung	19 (48.7)	9 (81.8)	10 (35.7)	0.014
Left lung	19 (48.7)	9 (81.8)	10 (35.7)	0.014
Bilateral	13 (33.3)	8 (72.7)	5 (17.9)	0.002
Lung lobe infiltrate (*n* (%))				
Right upper lobe	9 (23.1)	5 (45.5)	4 (14.3)	0.085
Right middle lobe	6 (15.4)	4 (36.4)	2 (7.1)	0.042
Right lower lobe	18 (46.2)	9 (81.8)	9 (32.1)	0.011
Left upper lobe	8 (20.5)	4 (36.4)	4 (14.3)	0.188
Left lower lobe	18 (46.2)	9 (81.8)	9 (32.1)	0.011
Interstitial inflammation (*n* (%))	2 (5.1)	1 (9.1)	1 (3.6)	0.490
Nodules (*n* (%))	8 (20.5)	3 (27.3)	5 (17.9)	0.401
Patchy infiltrate (*n* (%))	4 (10.3)	1 (9.1)	3 (10.7)	0.687
Fibrous proliferation (*n* (%))	15 (38.5)	4 (36.4)	11 (39.3)	1.000
Strip shadow (*n* (%))	2 (5.1)	2 (18.2)	0	0.074
Pleural effusion (*n* (%))	19 (48.7)	8 (72.7)	11 (39.3)	0.082
Treatment (*n* (%))				
Antiviral therapy	28 (71.8)	7 (63.6)	21 (75.)	0.368
Antibiotics	36 (92.3)	9 (81.8)	27 (96.4)	0.187
Corticosteroids	13 (33.3)	3 (27.3)	10 (35.7)	0.458
Immunoglobulin	6 (15.4)	2 (18.2)	4 (14.3)	0.553
Mechanical ventilation	11 (28.2)	2 (18.2)	1 (3.6)	0.187
Clinical prognosis (*n* (%))				
Severe pneumonia	7 (7.9)	3 (27.3)	4 (14.3)	0.379
Mortality	3 (7.7)	3 (27.3)	0	0.018

WBC, white blood cell; Hb, haemoglobin; Plt, platelet; CRP, C-reactive protein; PCT, procalcitonin; ALT, alanine aminotransferase; AST, aspartate aminotransferase; TP, total protein; Che, cholinesterase; Cr, creatinine.

Antiviral therapy includes ribavirin, acyclovir, ganciclovir and oseltamivir.

### Radiographic findings

All patients were examined by chest computed tomography (CT). In total, 64.1% (25/39) patients had lung inflammatory infiltration on radiographic images. The incidence of a right lung infiltrate was 81.8% (9/11) in the immunocompromised group, which was obviously higher than the 35.7% (10/28) in the immunocompetent group (*P* = 0.014). 23.1% (9/39) patients had upper lobe infiltrate, 15.4% (6/39) patients had middle lobe infiltrate and 46.2% (18/39) patients had lower lobe infiltrate in the right lung. Among those, 45.5% (5/11) of immunocompromised RAI patients had upper lobe infiltrate as opposed to 14.3% (4/28) of immunocompetent RAI patients (*P* = 0.085). Meanwhile, 36.4% (4/11) of the immunocompromised had middle lobe infiltrate as opposed to 7.1% (2/28) of the immunocompetent (*P* = 0.042), 81.8% (9/11) of the immunocompromised had lower lobe infiltrate as opposed to 32.1% (9/28) of the immunocompetent (*P* = 0.011).

A left lung infiltrate was present in 81.8% (9/11) in the immunocompromised group and 35.7% (10/28) in the immunocompetent group (*P* = 0.014). There were 36.4% (4/11) immunocompromised patients who had left upper lobe to infiltrate compared to 14.3% (4/28) immunocompetent patients (*P* = 0.188). The rate of left lower lobe infiltrate was 81.8% (9/11) in the immunocompromised group, which was obviously higher than the 32.1% (9/28) in the immunocompetent group (*P* = 0.011).

Other radiographic findings included interstitial inflammation, nodules, patchy infiltrate, fibrous proliferation and pleural infiltrate, which had no statistical significance between the two groups (Table 2).

**Fig. 1.**
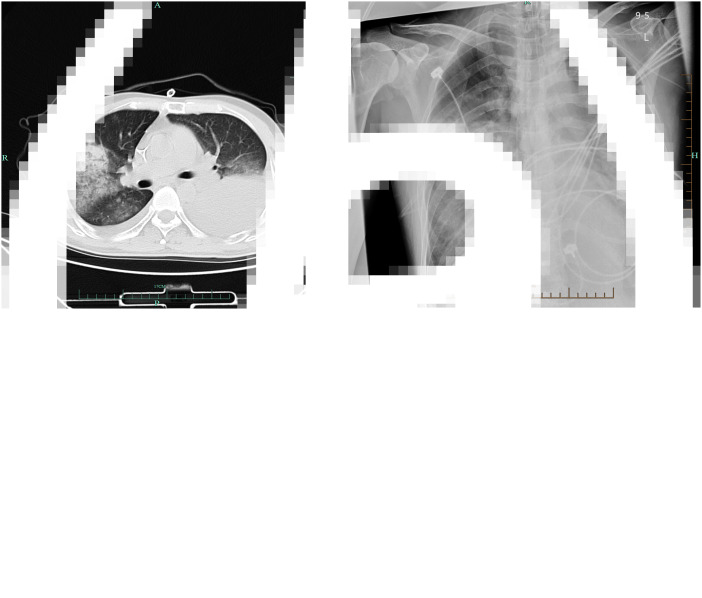
Radiological imaging of an immunocompromised RAI patient. (a) A ground glass changing on the middle lobe of the right lung and pleural effusion on the left lung on CT (11 October 2019). (b) Diffused and progressive bilateral infiltrates on chest X-ray (14 October 2019).

A total of 72.7% (8/11) immunocompromised RAI patients and 17.9% (5/28) immunocompetent RAI patients had bilateral infiltrates (*P* = 0.002) (Figure 1).

### Therapy and outcome

Fever, cough, sputum production and increased CRP and PCT led to antibiotics use. Empirical antiviral therapy, including ribavirin, acyclovir, ganciclovir and oseltamivir, was administered to 71.8% (28/39) of the patients. Corticosteroids (33.3%, 13/39), immunoglobulin (15.4%, 6/39) and mechanical ventilation (28.2%, 11/39) were also administered to patients. No significant difference for each treatment was found between the immunocompromised group and immunocompetent group (antibiotics: *P* = 0.368; antiviral therapy: *P* = 0.187; corticosteroids: *P* = 0.458; immunoglobulin: *P* = 0.553; mechanical ventilation: *P* = 0.187) (Table 2).

There were 17.9% (7/39) patients with severe pneumonia (CURB-65 scores ≥3), including 27.3% (3/11) immunocompromised RAI patients and 14.3% (4/28) immunocompetent RAI patients (*P* = 0.379). The overall mortality rate was 7.7% (3/39). Strikingly, the hospitalized mortality rate was 27.3% (3/11) among the immunocompromised RAI patients *vs.* zero among the immunocompetent RAI patients (*P* = 0.018) (Table 2).

## Discussion

The different manifestations of adenovirus infection between immunocompetent patients and immunocompromised patients are rarely discussed. Our current research suggests the following. (1) There was no statistical significance in clinical symptoms between the two groups. (2) Compared to immunocompetent patients, immunocompromised patients had a higher incidence of anaemia, thrombocytopaenia, hypoalbuminaemia, hyponatraemia, low levels of cholinesterase and high incidence of concomitant microorganisms in respiratory samples. (3) Immunocompromised patients had more involvement of the bilateral lungs and the lower lobes of both lungs. (4) Immunocompromised patients had higher CURB-65 scores and higher mortality rates than immunocompetent patients.

Immunocompromised RAI patients had a high incidence of anaemia, thrombocytopaenia, hypoalbuminaemia, hyponatraemia and low cholinesterase levels in our study. We speculated that these abnormal laboratory test results were partly related to adenovirus infection and partly related to their underlying diseases. For example, an adenovirus infection could result in hepatitis and pancytopenia [11]. Therefore, it is reasonable that hypoalbuminaemia, anaemia and thrombocytopaenia could present in RAI patients. Anaemia and thrombocytopaenia are also related to poor outcomes of viral pneumonia [12, 13]. Furthermore, anaemia is a prognostic indicator for liver transplantation, AIDS and cancer [14–16].

Hypoalbuminaemia is an independent predictor of mortality in both compensated and decompensated cirrhosis, AIDS, diabetes and chronic kidney disease [17–20]. Previous studies indicated that serum cholinesterase is an important clinical marker of inflammation and reduced serum cholinesterase indicates severe systemic inflammation in critically ill patients [21, 22]. Decreased serum albumin and cholinesterase could be explained by poor appetite and predisposing conditions leading to reduced intake, decreased synthesis and increased usage of proteins [23].

Serum sodium concentration is recognised as a prognostic factor in liver cirrhosis, cancer and AIDS patients [24–26]. Our study showed that the serum sodium concentration was lower in the immunocompromised group than in the immunocompetent group. It was noted that inappropriate secretion of antidiuretic hormone was found in adenovirus infected patients, which was associated with hyponatraemia [27].

Co-infection was often reported both in immunocompetent and immunocompromised individuals [7, 10, 28, 29]. Concomitant infections in immunocompromised patients tend to deteriorate into severe pneumonia and even accelerate progress to death [3, 30]. The immunocompromised had a higher co-incidence of other microorganisms in respiratory samples than the immunocompetent (*P* = 0.017).

Several studies indicated that the initial radiographic findings of RAI were similar to those of bacterial pneumonia [30–32]. A review reported that patchy, diffuse infiltrates, consolidation and occasionally pleural effusion were major radiographic findings [6]. It seems that there are no specific radiographic characteristics to distinguish an adenovirus infection from other microbe infections. Update, few researchers have focused on radiographic characteristics of RAI patients with different immune statuses. A retrospective study in paediatric patients indicated that bilateral infiltrate was observed in 63% of patients and multifocal involvement was in 68% of patients. Furthermore, the left lower and right upper lobes were prone to be involved [33]. Meanwhile, another study showed that lower lobe involvement was commonly observed in adult RAI patients [34].

Consistent with the results of previous studies, our data showed 72.7% (8/11) of immunocompromised patients presented with bilateral lung infiltrate as opposed to 17.9% (5/28) in the immunocompetent group (*P* = 0.011). The lower lobes of both lungs in the immunocompromised group were more vulnerable to infiltration than those in the immunocompetent group. The bilateral and multifocal involvement might be associated with predisposing diseases. We speculated that pathogens were easier to spread in lungs under impaired immune conditions.

Although antiviral drugs were empirically used, whether antiviral drugs improve clinical outcomes in the context of RAI remains controversial. Most experts believed that no medicines have been proven effective to treat adenovirus infection [2, 7, 35]. However, few studies indicated that earlier initiation of treatment might be associated with favourable clinical outcomes in immunocompromised RAI patients [36, 37].

Our present study suggested that the prognosis of RAI patients was associated with immune status. A total of 27.3% (3/11) of immunocompromised patients with severe pneumonia died, whereas 14.3% (4/28) of immunocompetent patients with severe pneumonia eventually recovered during the follow-up. The clinical characteristics of RAI in immunocompromised patients can vary from mild infections to severe conditions [32]. It was noted that RAI usually followed a fatal course in people with impaired immunity [38]. Underlying diseases also make immunocompromised patients susceptible to other pathogens [3]. Therefore, clinicians should consider the possibility of adenovirus infection in severe pneumonia patients with negative cultures and/or poor response to antibiotics.

There were several limitations of our study. (1) In our retrospective study, we did not examine adenovirus types in the respiratory and blood samples, nor did we explore the relationship between different virus types and the clinical outcomes. It was reported that different serotypes had different clinical manifestations and outcomes [1, 2]. We also did not carry out viral load tests in peripheral blood or respiratory samples to further determine the potential of the viral load to predict the outcomes. (2) Our study was based on small sample size, and additional studies based on a large population are necessary to verify our findings. (3) The effects of underlying diseases on clinical presentations and radiographic features were not fully evaluated.

## Conclusion

In summary, our data demonstrated that immunocompetent and immunocompromised adult RAI patients displayed obviously different clinical features and outcomes. In particular, mortality in immunocompromised RAI patients was significantly higher than that in immunocompetent patients.

## Data Availability

The data that support the findings of this study are included in our manuscript.
